# Contact Tracing during an Outbreak of Ebola Virus Disease in the Western Area Districts of Sierra Leone: Lessons for Future Ebola Outbreak Response

**DOI:** 10.3389/fpubh.2016.00130

**Published:** 2016-06-22

**Authors:** Olushayo Oluseun Olu, Margaret Lamunu, Miriam Nanyunja, Foday Dafae, Thomas Samba, Noah Sempiira, Fredson Kuti-George, Fikru Zeleke Abebe, Benjamin Sensasi, Alexander Chimbaru, Louisa Ganda, Khoti Gausi, Sonia Gilroy, James Mugume

**Affiliations:** ^1^World Health Organization (WHO), Kigali, Rwanda; ^2^World Health Organization (WHO), Geneva, Switzerland; ^3^World Health Organization (WHO), Kampala, Uganda; ^4^Ministry of Health and Sanitation, Freetown, Sierra Leone; ^5^Western Area District Health Management Team, Freetown, Sierra Leone; ^6^World Health Organization (WHO), Freetown, Sierra Leone; ^7^WHO Intercountry Support Team for Eastern and Southern Africa, Harare, Zimbabwe; ^8^United Nations Population Fund, Freetown, Sierra Leone

**Keywords:** Ebola virus disease, outbreak response, contact tracing, surveillance, Western Area, Sierra Leone, research article

## Abstract

**Introduction:**

Contact tracing is a critical strategy required for timely prevention and control of Ebola virus disease (EVD) outbreaks. Available evidence suggests that poor contact tracing was a driver of the EVD outbreak in West Africa, including Sierra Leone. In this article, we answered the question as to whether EVD contact tracing, as practiced in Western Area (WA) districts of Sierra Leone from 2014 to 2015, was effective. The goal is to describe contact tracing and identify obstacles to its effective implementation.

**Methods:**

Mixed methods comprising secondary data analysis of the EVD case and contact tracing data sets collected from WA during the period from 2014 to 2015, key informant interviews of contact tracers and their supervisors, and a review of available reports on contact tracing were implemented to obtain data for this study.

**Results:**

During the study period, 3,838 confirmed cases and 32,706 contacts were listed in the viral hemorrhagic fever and contact databases for the district (mean 8.5 contacts per case). Only 22.1% (852) of the confirmed cases in the study area were listed as contacts at the onset of their illness, which indicates incomplete identification and tracing of contacts. Challenges associated with effective contact tracing included lack of community trust, concealing of exposure information, political interference with recruitment of tracers, inadequate training of contact tracers, and incomplete EVD case and contact database. While the tracers noted the usefulness of community quarantine in facilitating their work, they also reported delayed or irregular supply of basic needs, such as food and water, which created resistance from the communities.

**Conclusion:**

Multiple gaps in contact tracing attributed to a variety of factors associated with implementers, and communities were identified as obstacles that impeded timely control of the EVD outbreak in the WA of Sierra Leone. In future outbreaks, early community engagement and participation in contact tracing, establishment of appropriate mechanisms for selection, adequate training and supervision of qualified contact tracers, establishment of a well-managed and complete contact tracing database, and provision of basic needs to quarantined contacts are recommended as measures to enhance effective contact tracing.

## Introduction

Sierra Leone experienced a major, unprecedented outbreak of Ebola virus disease (EVD) from early 2014 to late 2015. With a total of 8,704 confirmed cases (accounting for 57% of all confirmed cases in West Africa), 3,589 deaths from confirmed cases and a case fatality rate of 41.2%,^1^ the country was classified as having intense transmission of EVD. All districts in Sierra Leone were affected by the outbreak at various times. The Western Area (WA), one of the four administrative divisions of Sierra Leone, which encompasses urban and rural districts, the most populated of the 14 administrative districts in Sierra Leone, experienced intense transmission of EVD between June 2014 and August 2015, when the last confirmed case died at the Ebola treatment center (1). The district accounted for an estimated 40% of all confirmed cases reported in Sierra Leone during the outbreak.^2^

WA has a landmass of 557 km^2^ and an estimated population of 1,304,507.^3^ It is subdivided into 49 urban and 20 rural wards. It is the main economic and financial center of the country, and therefore it has a transient population, with a high influx of people from rural communities from all parts of the country arriving in search of economic opportunities and better living standards. The area has several urban slums and informal trading (and fishing) settlements, which are densely populated and have poor living conditions.

EVD is a highly infectious zoonotic disease caused by the Ebola virus. Primary infection occurs when infected animals, such as fruit bats and non-human primates, transmit the disease to humans, while secondary transmission is human-to-human, through close contact with body fluids of a sick person, body of a deceased patient, or contaminated environments (2, 3). The pattern in Sierra Leone, including in WA, was human-to-human transmission. Because of the mode of transmission, the World Health Organization (WHO) recommends contact tracing as one of the critical interventions for prevention and control of EVD during outbreaks.

Contact tracing involves systematic identification and recording of all persons exposed to a symptomatic Ebola patient (classified as a probable or confirmed case), their infected body fluids or corpse, and monitoring them daily for EVD symptoms for a period of 21 days, from the last day of exposure.^4^ Contact tracing during outbreaks of EVD, coupled with active case search and alert, case investigation, laboratory testing, and database analysis, constitutes the surveillance and epidemiological investigation component of the EVD control strategy (see text footnote 3). The principles of contact tracing are based on the premise that during outbreaks of EVD, secondary chains and other new cases normally arise from contact with infected patients (see text footnote 3). Therefore, contact tracing ensures that all exposed persons, who are likely to develop symptoms of the diseases, are monitored daily during the incubation period, and those who become symptomatic are immediately isolated, therefore breaking the chain of transmission (see text footnote 3). Other EVD control measures include isolation, management and treatment of patients, strict infection prevention and control, safe and dignified burial of the dead, and community mobilization and engagement (4–6).

Recent scientific evidence suggests that the intense transmission of EVD, the prolonged duration of the outbreak, and the high case load in the West Africa outbreak are, to some extent, due to poor contact tracing (7, 8). Anecdotal evidence also attributed the high caseload and inability to promptly contain the outbreak to a high population density, overcrowding, highly mobile populations, and several unregistered informal settlements, which could have provided fertile ground for sustained transmission of the disease (9). These factors further complicated effective contact tracing and overall outbreak prevention and control.

In this study, we describe contact tracing as it was performed in an urban area of Sierra Leone, from June 2014 to August 2015. We answered the research question as to whether contact tracing as performed in WA was effective or not, and what were the associated issues and challenges during the study period? The objectives of the study were to understand the characteristics, effectiveness, and challenges of contact tracing in WA and to propose appropriate recommendations for improving EVD contact tracing during future outbreaks, with a focus on urban settings.

## Materials and Methods

### Study Design

We conducted a descriptive study of the EVD contact tracing activities that were implemented from June 2014 to August 2015 in WA, Sierra Leone. A mixed methods approach was used, including secondary data analysis, key informant interviews, and a review of existing reports. Secondary data analysis was conducted on the EVD case and contact tracing data for WA, generated as part of the epidemiological investigations during the 2014/2015 EVD outbreak response. Key informant interviews, which sought to establish facts on the implementation, evolution, and challenges associated with implementing contact tracing activities, were conducted with key persons, including contact tracers and contact tracing supervisors, who were directly or indirectly involved in contact tracing activities. We also reviewed available reports on contact tracing in WA.

### Data Collection and Analyses

In Sierra Leone, active EVD case search and identification as well as the identification and listing of contacts were aided by the use of standardized EVD case definitions, categorized into suspect, probable, and confirmed cases (Table 1), and a standardized definition for an EVD contact. A contact was defined as “*a person who undertook unprotected care for a patient of Ebola or participated in the burial of an Ebola death or washed linen, bathed a patient, cleaned body fluids of a case or slept in the same room with a case*.”^5^

**Table 1:** **Definition of suspected, probable, and confirmed cases of EVD in Western Area districts, Sierra Leone: June 2014 to August 2015**.^1^

Case	Definition
Suspected	A suspected Ebola patient is any person presenting with acute fever (>38°C) and three or more of the following symptoms:
	• Headache • Loss of appetite • Fatigue • Difficulty breathing • Nausea • Difficulty swallowing • Vomiting • Hiccups • Diarrhea • Muscle or joint pain • Abdominal pain • Unexplained bleeding
	OR anyone who is ill and either:
	Cared for or was cared for by someone who had Ebola, Attended a funeral of someone with Ebola, In the case of a child, breastfed by a confirmed Ebola mother or caretaker
	OR any unexplained death
Probable	Any person meeting the suspected case definition criteria and has had contact (epidemiological link) with a confirmed case
	OR any unexplained death
Confirmed	A probable or suspected case whose laboratory test is positive for Ebola virus

^1^Case definition recommendations for Ebola or Marburg virus diseases. WHO. (2014) http://apps.who.int/iris/bitstream/10665/146397/1/WHO_EVD_CaseDef_14.1_eng.pdf?ua=1 [Accessed on May 16, 2016].

Case investigation and contact tracing forms are completed for every suspected or probable case of EVD investigated by the alert and response team, which comprise district surveillance officers and data clerks. The cases are then transferred to EVD holding centers/community care centers (CCC), where blood samples are taken and sent to the laboratory for testing. A copy of the case investigation form accompanies the blood sample to the laboratory, where a laboratory identification number is assigned. Once the laboratory results are received, the case investigation and contact tracing forms are updated accordingly and then entered into the viral hemorrhagic fever (VHF) software, which was developed by the United States Centers for Disease Control and Prevention (CDC)^6^ by district data clerks. All districts of the country share their data with the national EVD data management center in Freetown, who then aggregates them into a national database on a daily basis. Case and contact data are linked using unique case identification numbers in the EVD database.

For this study, we extracted all datasets for WA from the national EVD database that met the confirmed EVD case definition and all contacts for the period from June 2014 to August 2015. The data were cleaned and exported to Microsoft Excel, where descriptive analysis was conducted. The total number of confirmed cases and contacts, number of confirmed cases with linked contacts, number of contacts linked to the confirmed cases, and mean number of contacts per case were generated in the first stage of the analysis. In the second stage, we described the sociodemographic and epidemiological characteristics of contacts who were linked to cases in terms of place, person, time, relationship to case, and type of exposure.

Key informant interviews were conducted to generate qualitative data. A key informant interview guide, which explained the purpose of the study, objectives of the key informant interviews, selection criteria, and profile of interviewees, the interview process, and guiding questions, was developed. The interview guide had five main themes, which explored the field experiences of the contact tracers/surveillance officers, successes, challenges, ways forward for improving contact tracing, and usefulness of quarantine to contact tracing. The research team used a purposive sampling method to identify five contact tracers and five contact tracer supervisors from a list of contact tracers in WA. The selection criteria ensured a good mix of interviewees in terms of age, gender, and educational level. The key informant interview was administered by members of the research team. The responses were coded, organized by thematic area, and summary counts generated for each thematic area.

We also obtained and conducted desk reviews on daily and weekly epidemiological bulletins for WA and other relevant available field investigation reports and extracted relevant information pertaining to contact tracing in WA.

### Ethical Considerations

This study was conducted as part of the post-outbreak review of the outbreak response to provide evidence for improving contact tracing and EVD surveillance during future outbreaks. Ministry of Health and Sanitation (MOHS) and the WA district health management team approved the study, and informed consent was obtained from all the key informant interviewees. All data presented in this article are anonymous.

## Results

The first case (and contacts) of EVD was recorded in WA on June 21, 2014, and the last case died at the Ebola treatment center on August 11, 2015. During this period, a total of 3,838 confirmed cases and 32,706 contacts were reported in WA and listed in the VHF database (Table 2). The epidemic peaked in mid-November 2014, followed by a steep decline until April 2015.

**Table 2:** **Summary of confirmed EVD cases and contacts and related indicators in Western Area districts, Sierra Leone: June 2014 to August 2015**.

	Urban (%)	Rural (%)	Aggregated totals (%)
Total number of confirmed cases	2,495 (65.0)	1,343 (35.0)	3,838
Proportion of confirmed cases who were listed as contacts at the time of their illness	519 (20.8)	333 (24.8)	852 (22.1)
Total number of contacts^a^	16,319 (63.9)	9,232 (36.1)	32,706
Number of confirmed cases with linked contacts in database^b^	424 (17.0)	282 (21.0)	714 (18.6)
Number of contacts linked to confirmed cases^c^	9,726 (59.6)	3,167 (34.3)	16,042 (49.0)
Mean number of contacts per case	23	11	22 (8.5)

*^a^7,155 records without residence excluded from the analysis for Western area urban and rural*.

*^b^Eight records without address excluded from the analysis for Western area urban and rural*.

*^c^3,194 records with missing address excluded from the analysis for Western area urban and rural*.

**Figure 1:**
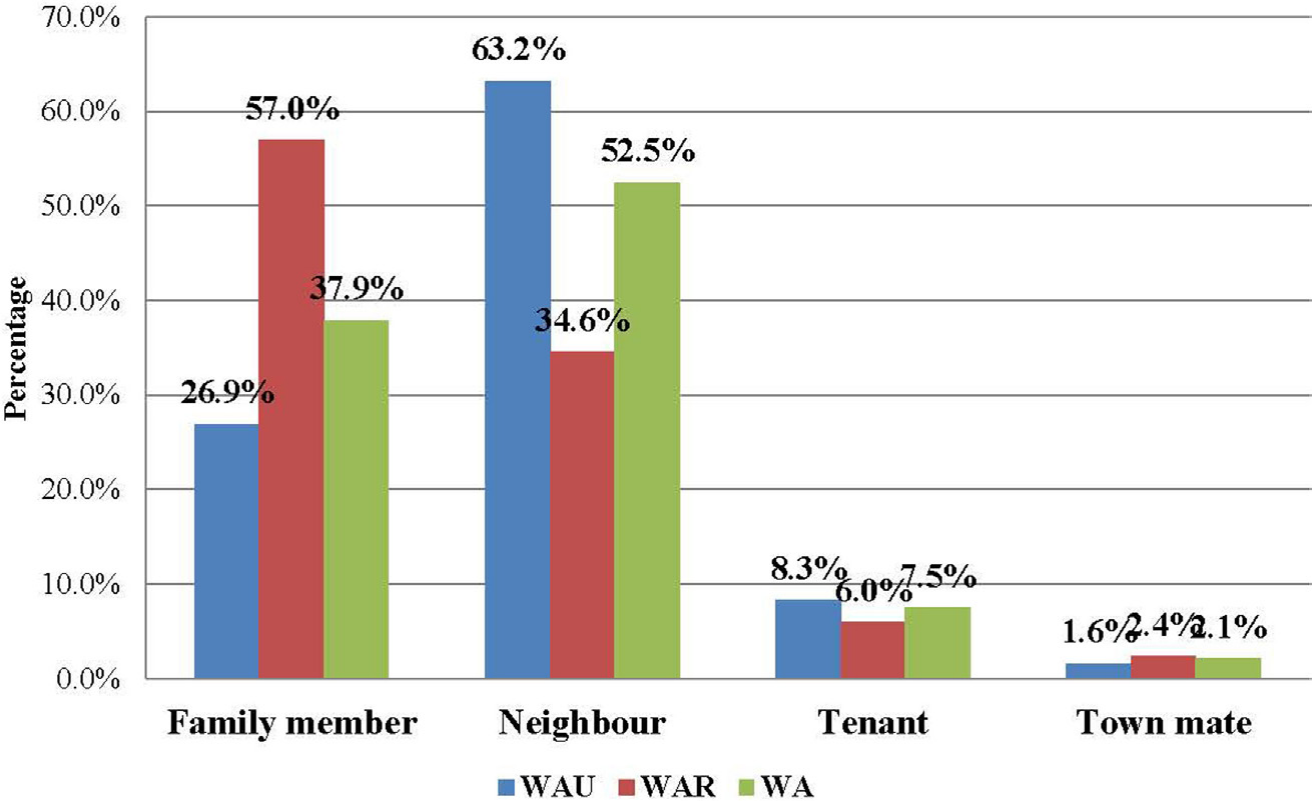
**Relationship of EVD contacts to cases in Western Area, Sierra Leone: June 2014 to August 2015 (*N* = 22,469)**.

The majority of the contacts in WA (more than 80%) were below 35 years of age, with the age groups 6–15 years (23.0%), 16–25 years (25.6%), and 26–35 years (17.9%) constituting the most affected people. The age distributions of contacts were similar in both districts. The youngest contact was 4 days old, the oldest was 109 years, and the mean age was 22.7 years. Just under half of the contacts (49.1%) were male, while females accounted for 50.9% (Table 3). Overall in WA, more than half of the contacts (52.5%) were neighbors of the confirmed case for which they were listed as contact, while 37.9% were family members. This trend is different between the two districts. In WA urban district, the majority of contacts (63.2%) were neighbors, whereas in the rural district, the majority (57.0%) were family members (Figure 1).

**Table 3:** **Demographic and exposure characteristics of EVD contacts in Western Area districts, Sierra Leone: June 2014 to August 2015**.

Contact characteristics	Age category	Urban (%)	Rural (%)	Total of WA (%)	Remarks
Age categories (years)	0–5	2,066 (13.1)	1,307 (14.5)	4,328 (13.9)	7,954 records excluded from the disaggregated data and 1,643 from the WA data
	6–15	3,663 (23.2)	2,168 (24.1)	7,427 (23)	
	16–25	4,178 (26.5)	2,227 (24.8)	7,960 (25.6)	
	26–35	2,916 (18.5)	1,561 (17.4)	5,559 (17.9)	
	36–45	1,504 (9.5)	854 (9.5)	2,949 (9.5)	
	46–55	736 (4.7)	461 (5.1)	1,470 (4.7)	
	55 and over	698 (4.4)	413 (4.6)	1,370 (4.4)	
	Mean	23	22.8	22.7	7,906 records excluded from the disaggregated data and 1,641 from the WA data
	Mode	25	25	25	
	Range	109	109	109	
Gender	Male	8,055 (49.6)	4,496 (49.1)	15,914 (49.1)	7,306 records excluded from the disaggregated data and 278 from the WA data
	Female	8,193 (50.4)	4,656 (50.9)	16,514 (50.9)	
Type of exposure	Touched body fluids of case (1)	123 (0.9)	29 (0.4)	163 (0.7)	11,925 records excluded from the disaggregated data and 10,614 from the WA data
	Had direct physical contact with body of case (dead or alive) (2)	4,814 (34.9)	1,210 (17.3)	6,121 (27.7)	
	Touched or shared the linen, clothes, or dishes/eating utensils of case (3)	816 (5.9)	85 (1.2)	954(4.3)	
	Slept, ate, or spent time in the same household or room as the case (4)	1,973 (14.3)	474 (6.8)	2,627 (11.9)	
	Combined (1) and (2)	1,134 (8.2)	247 (3.5)	1,397 (6.3)	
	Combined (1), (2), and (3)	277 (2.0)	394 (5.6)	730 (3.3)	
	Combined (1), (2), (3), and (4)	2,371 (17.2)	2,717 (38.8)	5,553 (25.1%)	
	Combined (1), (2), and (4)	86 (0.6)	16 (0.2)	107 (0.5)	
	Combined (1) and (3)	14 (0.1)	5 (0.1)	21 (0.1)	
	Combined (1), (3), and (4)	28 (0.2)	60 (0.9)	97 (0.4)	
	Combined (1) and (4)	61 (0.4)	7 (0.1)	68 (0.3)	
	Combined (2) and (3)	364 (2.6)	183 (2.6)	607 (2.7)	
	Combined (2), (3), and (4)	630 (4.6)	768 (11.0)	1,542 (7)	
	Combined (2) and (4)	455 (3.3)	108 (1.5)	602 (2.7)	
	Combined (3) and (4)	628 (4.6)	706 (10.1)	1,503 (6.8)	

**Figure 2:**
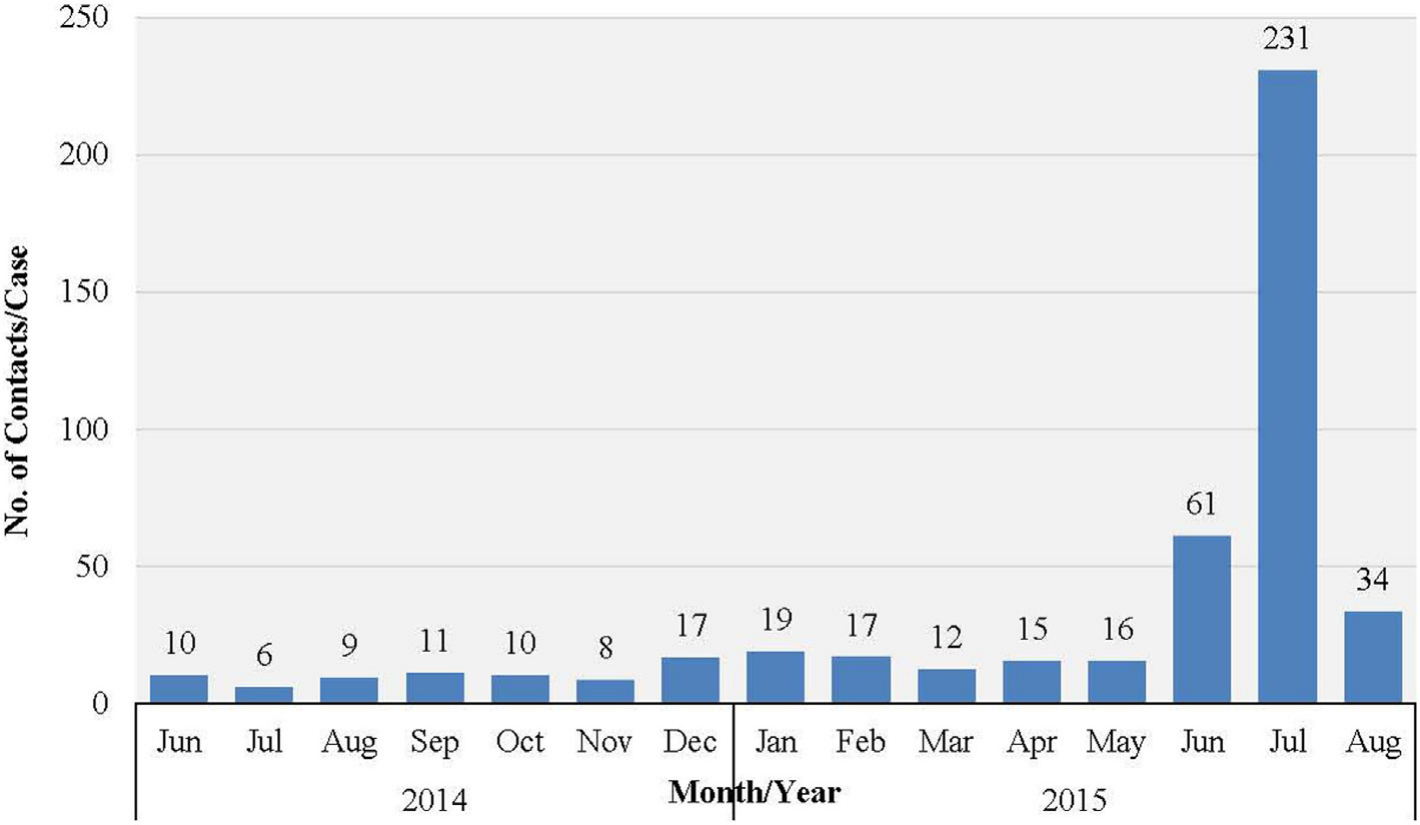
**Mean number of EVD contacts per case each month in WA, Sierra Leone: June 2014 to November 2015**.

**Figure 3:**
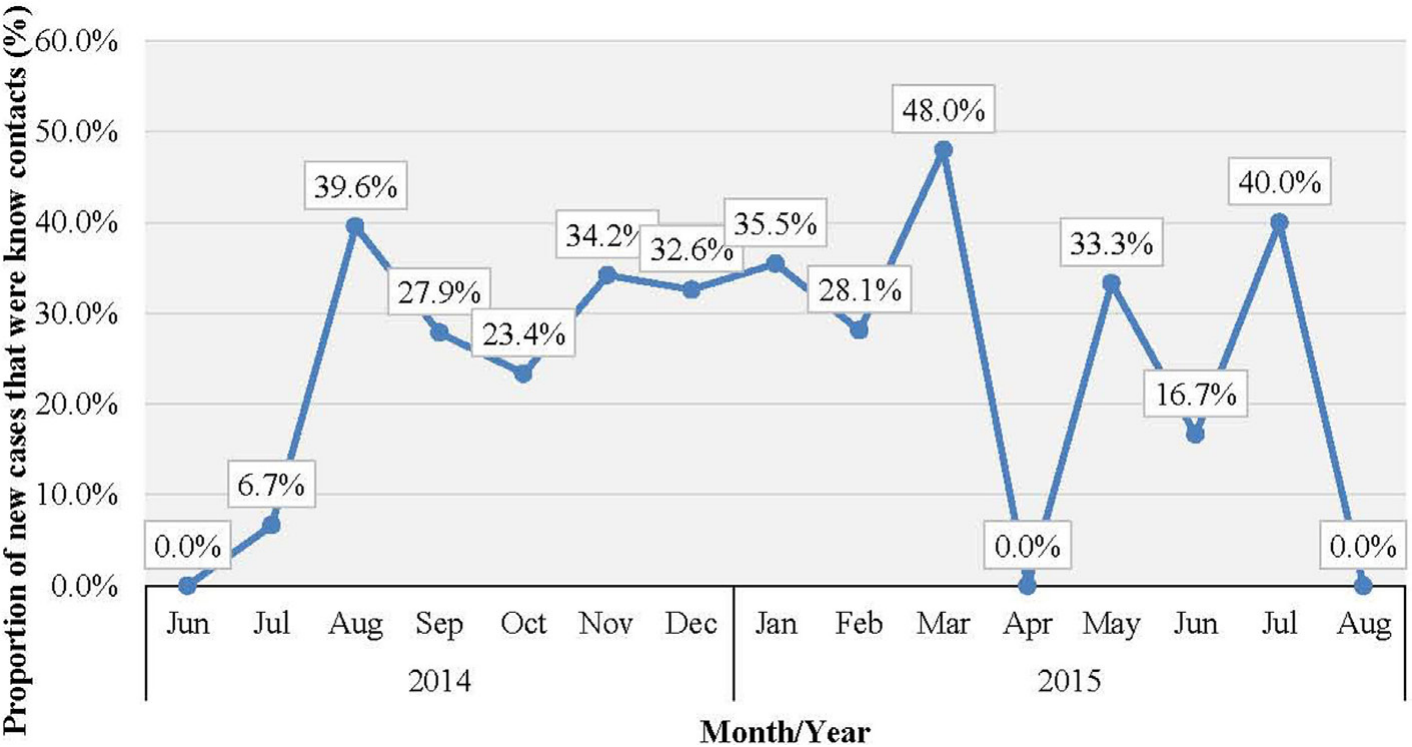
**Proportion (%) of new EVD cases who were known contacts in WA, Sierra Leone: June 2014 to August 2015**.

Western Area urban district accounted for the majority of confirmed cases (65.0%) and contacts (63.9%). In total, 852 (22.1%) of confirmed cases were reported to have come from the listed contacts, at the time of detection. Only 714 (18.6%) of confirmed cases had contacts directly linked to them in the EVD database. About half of all the listed contacts (49.0%, 16,042) were linked to the 714 confirmed cases (Table 2); the rest of the contacts were not directly linked to any confirmed cases in the EVD database. Based on the total number of listed confirmed cases and contacts, the mean number of contacts per case was 8.5. The mean number of contacts per case in WA urban district was twice that of the rural district (Table 2). The mean number of contacts per case ranged from 10 to 19 between June 2014 and May 2015; this rose significantly to 61 and 231, respectively, in June and July 2015 (Figure 2). The proportion of new EVD cases who were known contacts in WA fluctuated between 0.0 and 48.0% during the outbreak (Figure 3).

The most common type of exposure was direct physical contact with a living or dead body of a case (27.7%), sleeping, eating, or spending time with a case in the same room or household (11.9%), and multiple exposure that combined touching of body fluids, physical contact, touching linen, and eating/spending time together in the same room (25.1%). Touching body fluids and linen used by a case accounted for only 0.7 and 4.3% of exposures, respectively (Table 3).

Ten key informants were interviewed, including five contact tracers and five contact tracer supervisors. The majority of the interviewees mentioned timely identification of contacts who became cases as the main achievement in contact tracing. Many respondents reported that only up to 10.0% of the contacts monitored developed symptoms and became confirmed cases. One contact tracer summarized their success as “*we were able to finish the Ebola battle*.” Stigmatization and hostility to contact tracers, withholding of critical information on the status of contacts, provision of incorrect personal details, such as the name, address, and exposure of contacts, were identified as key challenges faced by contact tracers. *“Community trust was hard to gain”* reported one contact tracer. Many of the interviewees also mentioned late or irregular supply of food and water to contacts in quarantined homes as a challenge, which contributed to community resistance (Table 4). Almost all the interviewees agreed that community quarantining facilitated their work as contact tracers.

**Table 4:** **Summary of key information interviews: main achievements, challenges, and recommendations for improving EVD contact tracing in Western Area districts, Sierra Leone**.

	Key findings	Frequency
Successes achieved	Timely identification of contacts who became cases	9/10
	Estimated percentage of contacts who developed symptoms (%)	
	0–10	4/10
	11–20	1/10
	21–30	1/10
	More than 30	1/10
Challenges experienced	Stigmatization and hostility toward contact tracers	6/10
	Provision of wrong information about names and address and withholding of critical information on contacts	5/10
	Late or irregular supply of food and water to quarantined contacts	4/10
How to improve contact tracing	Ensure provision of appropriate supplies and equipment to contact tracers	7/10
	Better selection criteria for contact tracers	5/10
	Improve training, regular retraining and reorientation of contact tracers	4/10
	Provision of means of transport and communication and better remuneration of contact tracers	4/10
	Improved food and water supply to quarantined homes	2/10
	Improved data contact tracing data transmission using mobile technology	1/10
Quarantine to contact tracing	Community quarantining facilitated their work as contact tracers	8/10

The majority of the interviewees indicated that provision of appropriate supplies and equipment, such as thermometers, hand sanitizers, rain boots, and rain coats, to contact tracers would improve the contact tracing process during future outbreaks. One contact tracer remarked that “*the government should provide basic equipment and logistics to contact tracers*.” Many of the interviewees were of the opinion that better criteria for selection of contact tracers would ensure selection of good contact tracers and improve the quality of contact tracing. The interviewees proposed the following criteria for the selection of contact tracers: level of education, membership of the local community, and some background in health. Many of the interviewees also emphasized the importance of ensuring that the selection process is devoid of political interference. One contact tracer said “*a contact tracer should be selected and trained at the ward level*,” while another said “*let them work within their own communities and let there be no political interest*.” Many of the interviewees also mentioned provision of adequate training and regular retraining of contact tracers, provision of means of transportation, better remuneration for contact tracers, and more regular supply of food and water to quarantined homes as ways to improve contact tracing in future outbreaks. One contact tracer said contact tracers should be “*provided with feedback on the cases they identify and send to holding centers*” (Table 4).

## Discussion

Ebola virus disease outbreaks constitute major threats to global health security. Efficient and effective contact tracing, coupled with timely isolation of those who become symptomatic, is critical in limiting the spread and containing an outbreak. Until recently, EVD contact tracing had been confined to rural settings,^7^ where identification, listing, and follow-up of contacts was relatively easy (10). Transmission of EVD in urban settings (including a capital city), such as WA during the 2014/15 outbreak, presented new challenges (11) and realities, which would inform contact tracing strategy development in future outbreaks. It is therefore pertinent to understand the dynamics and challenges associated with contact tracing, especially in urban settings.

This study highlights weaknesses that were inherent in the quality and effectiveness of contact tracing activities in WA during the EVD outbreak. The findings of the study show that more than 75.0% of the new cases in WA were not listed as contacts at the time of their illness, and only 18.6% of confirmed cases in the database were linked to contacts; the proportion of identified contacts linked to cases was <50%. These poor indicators of performance of contact tracing activities demonstrate that contact identification and listing was neither comprehensive nor exhaustive. The missed EVD contacts are potential new chains of transmission, which may have contributed to the sustained transmission of EVD in WA (8). We identified provision of incorrect personal identity, lack of community trust in EVD prevention and control interventions (resulting in community resistance), and withholding of vital information on potential contacts and their health status as the challenges responsible for this trend; these findings are similar to those of other studies on contact tracing. Greiner et al. and Dixon et al. identified community misperception, fear, stigma, provision of incorrect personal identity, and the need to continue financial and social transactions as challenges to effective contact tracing (7, 8).

Perhaps, one of the most important factors associated with the inability to list all contacts is the stigma associated with being listed as a contact (7, 8). “*Sometimes they drove us out of their houses or just ran away on seeing us*” said one of the key informant interviewees. This results in movement of contacts to areas where they are unknown, therefore establishing multiple foci for transmission. The age distribution of the contacts shows that most of them belong to the economically productive age group. Although most of the key informant interviewees said that the quarantine policy facilitated their work, the policy may have prevented the productive age group from engaging in economic activities for at least 21 days, which is another disincentive for being listed as a contact (7, 8). In communities such as those in WA, where poverty levels are high (estimated to be 70.0%),^8^ it is important to provide for the needs of contacts of EVD cases during the period of monitoring and confinement, to encourage cooperation and collaboration from affected communities (12).

The overall mean number of contacts per patient recorded in this outbreak (8.5 contacts per patient) falls short of what was obtained in similar settings, such as Lagos, Nigeria (22.6 contacts per patient), Port Harcourt, Nigeria (133 contacts per patient), and Pujehun, Sierra Leone (11.5–25 contacts per patient) (13, 14), which further suggests underreporting of contacts. Given the very high population density in the urban slums and informal settlements of WA, we believe that the mean contacts per patient should have been much higher. In the Ebola outbreak in Nigeria, contact identification and listing was comprehensive, and over 95% of contacts were followed up on a daily basis. As a result, any contact who developed symptoms was isolated from the community immediately, and this contributed to the timely control of the EVD outbreak in that country. Perhaps, this was possible because of the limited magnitude of the outbreak, and the highly trained staff that implemented contact tracing.

In WA, the huge number of confirmed cases (and contacts) overwhelmed the EVD contact tracing system; the limited number of contact tracers could not cope with the workload, and this compromised the quality of the contact tracing process. Unpublished observations of contact tracing practices and reports during the outbreak showed that massive recruitment of contact tracers, which was fraught with political interference, recruitment of unsuitable (and often unmotivated contact tracers), and inability to agree on standardized procedures for contact tracing also compromised the quality of contact tracing practices in WA. For instance, the use of thermometers by contact tracers was widely debated at the national and district levels but never finally agreed upon, and therefore this practice was not uniformly applied in all areas.

In this study, the proportion of male contacts is almost equal to those who were female, which is similar to the findings from a similar study in Guinea (8). Regarding the relationship of the contacts to their source cases, neighbors comprised the highest percentage of contacts, followed by family members. However, a different pattern was observed between the urban and rural districts. In the rural district, the majority of the contacts (57.0%) were family members, while in the urban district, the majority (63.2%) were neighbors; these findings are similar to those of a contact tracing study in Guinea, where more than 50% of contacts in a rural area were observed to be family members of the source patients (8). This could be explained by social norms of the Sierra Leonean society, where families often live together (in family compounds) in rural areas, but not in urban areas. This finding should better inform contact identification and listing during future outbreaks in urban settings.

Physical contact with the body of a patient (dead or alive) and sleeping, eating, or spending time with patients were the most common types of contact exposure in WA; this finding is similar to that of other setting (13). In Sierra Leonean communities, taking care of sick relatives, friends, and neighbors and participation in funeral rituals involving touching dead bodies are widely practiced. These could have accounted for this trend. This highlights the importance of safe and dignified burial as a critical intervention in the control of EVD.

The sharp increase in the number of contacts per case reported in June/July 2015 was attributed to the additional measures of quarantining an area with a newly confirmed case, which was undertaken by national authorities at the height of the outbreak. This enabled monitoring of the whole population under quarantine in order to prevent potentially unidentified high-risk contacts from escaping to another district. There was no clear trend in the number of new EVD cases who were known contacts during the study period. Normally, this number increases as the quality of contact tracing improves over time, which again highlights weaknesses in contact identification and tracing in WA.

This study also revealed weaknesses in the EVD contact database management system. Ideally, every contact must be linked to a source case; however, in this study, several contact listing forms had missing information on many variables, and 51.0% of the contacts were not linked to their source patient. Only 56.8% of urban contacts and 34.3% of rural contacts were linked to a source case, while more than 80% of confirmed cases had no linked contact in the EVD contact database. This is a potential challenge for effective monitoring of the contact tracing component of the EVD outbreak response. Furthermore, our findings showed no evidence for the use of the contact tracing database for decision making.

### Study Limitations

This study is subject to three main limitations. The first is the incompleteness and quality of the VHF database; a significant proportion of the confirmed cases were not linked to specific contacts. Furthermore, some of the records had missing information on important variables, such as residence and relationship of contact to case values, which made disaggregation of the data by district challenging. Some of the values for variables, such as age, had outliers that were not possible. This limitation raised potential data quality and validity challenges, which we addressed by cleaning the database and excluding records with incomplete or outlying data and contacts who were not linked to any confirmed case in the database.

The second limitation was associated with the interviewer bias during the key informant interviews. The involvement of many of the authors in the organization, supervision, and monitoring of contact tracing activities at various times may have introduced bias into these interviews, as they may have only focused on information that confirmed their predetermined perceptions. To address the problem of interviewer bias, the key informant interviewers were carefully selected from among the authors and appropriately trained on how to conduct the interviews.

The third limitation is interviewee bias, which is an intrinsic limitation of key informant interviews. The key informants may have preconceived perceptions of what the interviewers wanted to hear. To reduce this limitation, we ensured careful selection of key informants, taking into consideration factors, such as age, gender, education level, religion, ethnicity, and areas of assignment. Furthermore, their responses were cross checked with findings of available reports on lessons learnt on contact tracing in WA.

## Conclusion

Although achievements were recorded in contact tracing in WA during the study period, several challenges constraining effective contact tracing were observed. Our findings confirm the results of other studies (7, 12, 15) and identified incomplete identification and listing of contacts, due to a lack of disclosure of contact identification and history and inconsistent contact follow-up methods resulting in failure to reach and effectively monitor all contacts regularly, as critical challenges facing contact tracers. Inappropriate selection criteria and inadequate refresher training of contact tracers and lack of reliable contact tracing data were also identified as challenges impeding timely control of the outbreak in the WA of Sierra Leone. Our study also shows that in rural areas, family members are more likely to be contacts, while in urban areas, neighbors are more likely to be contacts. Furthermore, our findings demonstrate that physical contact with the body of patients (dead or alive) and sleeping, eating, or spending time with patients are the most plausible type of contact exposure. These findings shed more light on the dynamics of contact tracing and provide evidence that would inform more effective contact identification and listing during future outbreaks.

Based on our findings, we propose five main recommendations aimed at improving contact tracing during future outbreaks. First, establishment of appropriate mechanisms for selection of the right contact tracers, from local communities, would improve the quality of contact tracing. In this regard, mobilization, engagement, and participation of local communities in contact tracing activities should be encouraged (16) to obtain community collaboration, which would reduce resistance and facilitate voluntary provision of contact tracing information. This in turn would facilitate more comprehensive identification of contacts. Furthermore, participation of community leaders in the identification and listing of contacts should be encouraged as a means to facilitate more comprehensive listing of contacts. Second, provision of adequate training, retraining, and supervision of contact tracers is critical to ensure sustained quality of contact tracing interventions. Contact tracing methodology, interpersonal communication, and community engagement skills should be included in the standard package of such training. Third, establishment of a well-managed and complete contact tracing database, early in the course of an EVD outbreak would provide reliable data, which could then be used to monitor and evaluate the quality of contact tracing as well as guiding contact tracing decision-making. Fourth, timely provision of the necessary supplies, equipment, and means of transportation would be a good incentive to contact tracers. To facilitate this, guidance on the appropriate items, which should be adapted to the local context, should be adopted early in the response to improve the quality of contact tracing. Finally, in scenarios where contacts will be quarantined, it is important to cater for their basic needs, to encourage their collaboration and participation.

## Author Contributions

OO, ML, MN, BS, FD, and TS designed and coordinated the study. NS, FK-G, FA, OO, AC, LG, BS, JM, SG, ML, MN, and KG collected and analyzed the data. OO, ML, MN, KG, BS, LG, FD, TS, SG, JM, NS, FK-G, FA, AC, LG, and SG drafted the manuscript. All authors read and provided input on the final draft of manuscript and agreed to be accountable for all aspects of the work. FD, TS, and MN gave final approval for publication of manuscript.

## Conflict of Interest Statement

The authors declare that the research was conducted in the absence of any commercial or financial relationships that could be construed as a potential conflict of interest. The handling editor EC declared a past collaboration with the authors and the handling Editor states that the process nevertheless met the standards of a fair and objective review.

## Footnotes

^1^Ebola Situation Report – (16 December 2015). WHO (2015) http://apps.who.int/ebola/current-situation/ebola-situation-report-16-december-2015 [Accessed December 23, 2015].

^2^Government of Sierra Leone, Ministry of Health and Sanitation Ebola Virus Disease Situation Report (Sit-Rep) – 21 December 2015. MOHS. (2015) http://nerc.sl/sites/default/files/Ebola%20Situation%20Report_Vol%20572.pdf [Accessed on December 23, 2015].

^3^Ministry of Health and Sanitation, Sierra Leone. Sierra Leone Demographic and Household Survey 2013. MOHS. (2013) http://dhsprogram.com/pubs/pdf/FR297/FR297.pdf [Accessed on April 8, 2016].

^4^World Health Organization. Contact tracing during an outbreak of Ebola virus disease. Brazzaville: WHO. (2014) http://www.who.int/csr/resources/publications/ebola/contact-tracing-during-outbreak-of-ebola.pdf?ua=1 [Accessed on December 23, 2015].

^5^Emergency guideline: implementation and management of contact tracing for Ebola virus disease. WHO/CDC. (2015) http://apps.who.int/iris/bitstream/10665/185258/1/WHO_EVD_Guidance_Contact_15.1_eng.pdf?ua=1 [Accessed on March 4, 2016].

^6^The Epi Info Viral Haemorrhagic Fever Software. CDC. (2015) https://epiinfovhf.codeplex.com/ [Accessed on February 2, 2016 and May 5, 2016].

^7^WHO Ebola virus disease factsheet no 103. WHO. (2016) http://www.who.int/mediacentre/factsheets/fs103/en/ [Accessed on March 27, 2016].

^8^Acaps Sierra Leone Country Profile. Acaps. (2012) http://www.acaps.org/img/documents/c-acaps-country-profile-sierra-leone.pdf [Accessed on March 27, 2016].

## Abbreviations

CDC, US Centers for Disease Control; EVD, Ebola virus disease; MOHS, Ministry of Health and Sanitation; VHF, viral hemorrhagic fever; WA, Western Area.
